# A Few Good Reasons Why Species-Area Relationships Do Not Work for Parasites

**DOI:** 10.1155/2014/271680

**Published:** 2014-05-08

**Authors:** Giovanni Strona, Simone Fattorini

**Affiliations:** ^1^European Commission, Joint Research Centre, Institute for Environment and Sustainability, Via E. Fermi 2749, 21027 Ispra, Italy; ^2^Azorean Biodiversity Group (CITA-A) and Platform for Enhancing Ecological Research & Sustainability (PEERS), Department of Agrarian Sciences, University of Azores, Pico da Urze, Terceira, Azores, 9700-04 Angra do Heroísmo, Portugal

## Abstract

Several studies failed to find strong relationships between the biological and ecological features of a host and the number of parasite species it harbours. In particular, host body size and geographical range are generally only weak predictors of parasite species richness, especially when host phylogeny and sampling effort are taken into account. These results, however, have been recently challenged by a meta-analytic study that suggested a prominent role of host body size and range extent in determining parasite species richness (species-area relationships). Here we argue that, in general, results from meta-analyses should not discourage researchers from investigating the reasons for the lack of clear patterns, thus proposing a few tentative explanations to the fact that species-area relationships are infrequent or at least difficult to be detected in most host-parasite systems. The peculiar structure of host-parasite networks, the enemy release hypothesis, the possible discrepancy between host and parasite ranges, and the evolutionary tendency of parasites towards specialization may explain why the observed patterns often do not fit those predicted by species-area relationships.

## 1. Introduction

The species-area relationship (SAR), that is, the increase of species number with area, is one of the best-documented patterns in ecology [1, 2], yet it is debated if this relationship should follow a universal shape or if different systems should have different SAR models [3]. The conceptual framework of SAR studies has been also used to investigate host-parasite relationships, by identifying hosts as “areas” and parasites as species “inhabiting” these areas [4–6].

The SAR has been largely investigated in the context of the island biogeography theory and has been often explained by the random placement and the habitat diversity hypotheses. The random placement hypothesis states that the larger the area of an island, the higher the number of individuals inhabiting that area, and hence the number of species [7]. The habitat diversity hypothesis states that larger islands provide more habitats, which promotes species diversity [8, 9].

Extending these concepts to host-parasite systems, we can assume that hosts with broader distributions have greater chances to be reached by (and to establish symbiotic relationships with) a larger number of parasite species, which should lead to a positive relationship between host range size and associated parasite diversity (parasite-host range relationship (PHrR)). Similarly, we should expect that body size drives the number of parasite species per host, as more niches are available on larger hosts [5, 10] (parasite-host size relationship (PHsR)). Since host body size and host geographical range are often intercorrelated [11], one may expect that large sized and/or widely distributed host species should have a higher parasite diversity than small and/or narrowly distributed hosts [5, 6]. However, empirical studies focusing on these issues produced highly variable results [12, 13]. Some studies reported positive, but typically weak, relationships between host body size and parasite species richness [14, 15], whereas others found no correlation [5, 16–18]. In-line with the parallelism between host-parasite and species-area relationships, one may also speculate that hosts with large niche breadths should show a high parasite richness, due to the fact that they use a great variety of habitats and hence are exposed to a wide diversity of potential parasites. However, to our knowledge, this issue has never been addressed in the context of SAR analysis applied to parasitism and will not be further discussed in this paper.

The generally low value of host characteristics as predictors of parasite diversity led to the conclusion that “a given host trait correlates with parasite species richness in some studies but not others, and when it does, it explains only a small fraction of the variance in richness across different host species” [19]. This view, however, has been recently challenged by a meta-analysis [20], whose results suggest that host body size, geographical range size, and population density should be considered as universal predictors of parasite richness across host species. Moreover, the results provided in this meta-analysis suggest that this pattern should be valid regardless of the taxonomic group and study design [20].

The use of meta-analytic approaches has been however repeatedly indicated as producing inherently flawed results [21–23] and, according to Shapiro [21], the popularity of meta-analysis derives from its ability to “attain statistically stable estimates for effects of low magnitude.” Moreover, comprehensive meta-analytic studies that seem to provide definitive answers to scientific problems may have the negative effect of discouraging further research [21]. We fear that this is what is going to happen with the application of SAR meta-analysis to host-parasite systems.

Here we are not questioning the statistical correctness of the above-mentioned meta-analytic study [20]. Rather, we would like to highlight that the results presented in the original literature used in (and motivating) such study indicate that host features are, in fact, not strong predictors of parasite richness. We think that, even if a meta-analysis suggests the existence of general patterns, the value of the original studies that rejected the existence of those patterns should be not diminished. In particular, here we claim that the fact that many studies have found a lack of relationship between host size and parasite diversity deserves explanation. From this perspective, we can consider the expected positive PHrR and PHsR as the null hypotheses (parasite diversity is expected to increase with host size or distribution). In the following paragraphs we will discuss a few tentative explanations for the fact that these null hypotheses have been rejected in several case studies.

## 2. Network Structure

Host species and their parasites are arranged into antagonistic ecological networks [24, and references therein]. These networks are characterized by patterns such as nestedness, species cooccurrence and modularity, which are the result of the underlying processes that minimize competition and risk of coextinction [25–27]. For example, in a nested network, the set of parasite species using any host is a subsample of any richer set. This scenario, that seems to be ubiquitous in host-parasite systems [26], implies, in general, a good degree of variability in parasite species richness per host [28]. Although species range or body size may contribute to this variability, the number of parasites found on a given host can be regulated by other factors determining the network structure and particularly species interactions.

For example, a recent work demonstrated that specialist parasites minimize their risk of coextinction by using hosts with low vulnerability to extinction [27]. This pattern, which is closely related to the nested structure of antagonistic networks, may emerge from the fact that several features increasing vulnerability of a host may be inversely related to the probability of that host to establish a stable symbiotic relationship with a parasite [27]. Geographical range plays an important role in determining these relationships, but also several other fundamental aspects of host ecology may be involved, such as host population size (which may be negatively correlated with body size [29]) and persistence over evolutionary time [27]. This last aspect has been also suggested as an important determinant of parasite species richness (colonization time hypothesis [30]).

It should be also highlighted that large sized species are typically less abundant and more endangered than small sized ones [31], which may lead large sized hosts to harbour fewer parasite species than smaller sized ones, in contrast with PHsR expectations. What we are suggesting here is that the number of parasite species found on a host is determined by how much this host interacts with other hosts and that the degree of interactions can be affected by too many aspects of host ecology, biology, and ethology to be embedded into a general law.

## 3. Enemy Release

One of the reasons for the success of alien species is the absence of parasites in the newly colonized area [32–34]. Plant and animal invaders that escape their native enemies are less parasitized than conspecific populations in the native range [32, 35, 36]. Enemy release is due to the fact that most of the parasites that a colonist host might bring with it are either left behind during the colonization process, lost shortly thereafter, or cannot survive in the new area [37]. This effect may be partly counterbalanced by the fact that alien species tend to acquire local parasites in their new distribution areas [32]. However, some alien species, despite having acquired new parasites from native hosts, have been observed to be less parasitized than their native competitors (in terms of both parasite diversity and abundance) [38].

Despite the enemy release hypothesis has been addressed mainly to introduce species; it is based on principles that are applicable to any species capable of extending its range via jump dispersal. For example, the majority of fish species (and particularly reef fish) disperse as larvae, which may travel for very long distances [39] and are, in general, much less parasitized than adult individuals [40].

One of the main assumptions behind the application of SARs to host-parasite systems is that a host is likely to increase its parasitofauna through range expansion, due to the fact that a wide geographical range raises the odds of encountering new parasite species (PHrR). This assumption, however, is clearly in contrast with the fact that hosts may actually loose parasites during range expansion and that the acquisition of new parasite species from native hosts has been observed to be not sufficient to compensate such loss.

## 4. Sampling Biases

Our knowledge of parasite diversity is far from being complete [41], with most of the hosts being unsampled or heavily undersampled [42]. Parasitofaunas of hosts with wide distributions, which are also often more locally abundant [11], are probably better known than those of less common hosts. This should create artifactual evidence in support of the hypothesis that parasite richness is positively correlated with host geographic range (PHrR). To address this well-known problem, estimates of study efforts are usually introduced in parasitological studies [6, 43]. However, these corrections cannot solve another fundamental bias due to host spatial sampling. In general, host species have been screened for parasites only from a few localities within their distributional range. This may imply that we know only a small fraction of the parasite diversity associated with a certain host. Moreover, we cannot assume that the geographical distribution of a parasite equals that of its hosts. The actual ranges of parasite species may be indeed much more restricted than those of their known hosts, due to the fact that, as discussed in the previous paragraph, a host may lose some of its parasites during colonization [32, 35, 36].

This problem has no easy solution, and it is further complicated by difficulties in parasite identification. Host taxonomy has been long used to assist the identification of parasite species. Yet, the classification of several parasites has been recently challenged by new molecular evidence that enlightened how broadly distributed generalist parasites are indeed complexes of species [44], thus reinforcing the idea that parasite ranges are often much smaller than those of their suitable hosts and that SAR studies based on host parasite checklists may be indeed biased, unless detailed information on species distribution is provided along with host-parasite records.

## 5. Evolutionary Tendency Towards Host Specificity

**Figure 1:**
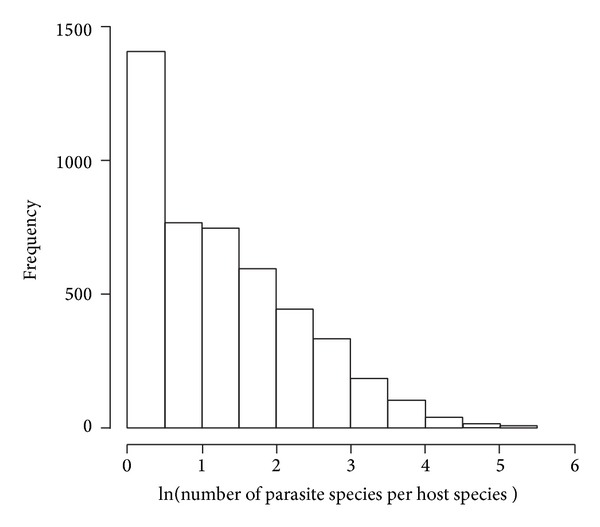
Histogram showing the distribution of the number of parasite species per host species obtained using all available data from FishPEST database (i.e., 33143 host parasite records, 4650 host species, and 11802 parasite species) [49–51]. The most recurrent situation is that of hosts used by only one parasite species.

Most parasites have few chances to find a proper final host, and infecting an unsuitable host would lead a parasite to starve, or to be killed by the host immune response, or to kill the host (and hence itself) [45]. Selective pressures would therefore favour two different, opposite evolutionary processes: parasites should become generalists or evolve more efficient behaviours to find hosts. Although the two strategies seem to draw a clear separation between generalist and host specific parasites, they overlap with various degrees, keeping parasite host ranges from being extremely narrow or extremely wide [46]. However, it is known that relatively few parasites use many fish hosts, which supports the hypothesis that adaptations enhancing host finding are evolutionarily more stable than those softening parasite niche requirements [27]. This skewed pattern might be even more pronounced because there are likely more cryptic species among parasites than among hosts. Host-parasite coevolution is one of the most commonly assumed causes for specialization [47] and parasites may have a tendency for overspecialization [48]. The fact that most parasites are species specific would therefore lead to a pronounced skew also in the distribution of the number of parasite species per host, with a few hosts harbouring several (specific) parasites and many hosts harbouring few (generalist) parasite species. This scenario, which is actually observed in available datasets (Figure 1), implies that (i) the number of parasite species found on a host tends to be low independently from the host body and/or range size (in contrast with PHhR and PHsR expectations), and (ii) a major factor affecting the number of parasites per host is parasite specificity itself, not host characteristics.

## 6. Concluding Remarks

Host-parasite interactions are far from being neutral [52]. This is the main reason why ecological approaches treating hosts as islands have been often criticized [53]. Randomness in encounters between hosts and parasites determines the odds for a symbiosis to be established: the more often a parasite meets a host, the higher its probability to success in host colonization. However, the capability of a parasite to expand its host range and the capability of a host to escape parasite infection are extremely variable not only at the species level but also at the individual one [54]. Thus, finding general laws describing parasite species richness is a difficult task [18]. Host body size and host range clearly play important roles in determining parasite richness, at least in setting its upper boundary: a small host would offer less physical space and niches to parasites, and a host with small distribution would have little chance to encounter parasites. Yet, we argue that the lower boundary, that is, the minimum number of parasites expected on a host with a given body size and a given geographical range, is affected by a much larger number of factors. In this paper we tentatively suggested a few of them. We hope that new studies will further investigate this issue, in order to shed light on the mechanisms making species-area relationships in host-parasite systems less common than expected in a merely neutral scenario.

## References

[1] Arrhenius O (1921). Species and area. *Journal of Ecology*.

[2] Rosenzweig ML (1995). *Species Diversity in Space and Time*.

[3] Williams MR, Lamont BB, Henstridge JD (2009). Species-area functions revisited. *Journal of Biogeography*.

[4] Lawton JH, Cornell H, Dritschilo W, Hendrix SD (1981). Species as islands: comments on a paper by Kuris et al. *The American Naturalist*.

[5] Nunn CL, Altizer S, Jones KE, Sechrest W (2003). Comparative tests of parasite species richness in primates. *The American Naturalist*.

[6] Poulin R, Guilhaumon F, Randhawa HS, Luque JL, Mouillot D (2011). Identifying hotspots of parasite diversity from species-area relationships: Host phylogeny versus host ecology. *Oikos*.

[7] Connor EF, McCoy ED (1979). The statistics and biology of the species-area relationship. *The American Naturalist*.

[8] McGuinness KA (1984). Equations and explanations in the study of species-area curves. *Biological Reviews*.

[9] Turner WR, Tjørve E (2005). Scale-dependence in species-area relationships. *Ecography*.

[10] Morand S, Guégan JF (2000). Distribution and abundance of parasite nematodes: ecological specialisation, phylogenetic constraint or simply epidemiology?. *Oikos*.

[11] Strona G, Galli P, Montano S, Seveso D, Fattorini S (2012). Global-scale relationships between colonization ability and range size in marine and freshwater fish. *PLoS ONE*.

[12] Poulin R (1997). Species richness of parasite assemblages: evolution and patterns. *Annual Review of Ecology and Systematics*.

[13] Poulin R (2004). Macroecological patterns of species richness in parasite assemblages. *Basic and Applied Ecology*.

[14] Ezenwa VO, Price SA, Altizer S, Vitone ND, Cook KC (2006). Host traits and parasite species richness in even and odd-toed hoofed mammals, Artiodactyla and Perissodactyla. *Oikos*.

[15] Bordes F, Morand S, Kelt DA, Van Vuren DH (2009). Home range and parasite diversity in mammals. *American Naturalist*.

[16] Feliu C, Renaud F, Catzeflis F, Hugot J-P, Durand P, Morand S (1997). A comparative analysis of parasite species richness of Iberian rodents. *Parasitology*.

[17] Krasnov BR, Shenbrot GI, Khokhlova IS, Degen AA (2004). Flea species richness and parameters of host body, host geography and host ‘milieu’. *Journal of Animal Ecology*.

[18] Korallo NP, Vinarski MV, Krasnov BR, Shenbrot GI, Mouillot D, Poulin R (2007). Are there general rules governing parasite diversity? Small mammalian hosts and gamasid mite assemblages. *Diversity and Distributions*.

[19] Poulin R, Forbes MR (2012). Meta-analysis and research on host-parasite interactions: past and future. *Evolutionary Ecology*.

[20] Kamiya T, O'Dwyer K, Nakagawa S, Poulin R (2014). What determines species richness of parasitic organisms? A meta-analysis across animal, plant and fungal hosts. *Biological Reviews*.

[21] Shapiro S (1994). Meta-analysis/Shmeta-analysis. *The American Journal of Epidemiology*.

[22] Feinstein AR (1995). Meta-analysis: statistical alchemy for the 21st century. *Journal of Clinical Epidemiology*.

[23] Sharpe D (1997). Of apples and oranges, file drawers and garbage: why validity issues in meta-analysis will not go away. *Clinical Psychology Review*.

[24] Dunne JA, Lafferty KD, Dobson AP (2013). Parasites affect food web structure primarily through increased diversity and complexity. *PLoS Biology*.

[25] Bastolla U, Fortuna MA, Pascual-García A, Ferrera A, Luque B, Bascompte J (2009). The architecture of mutualistic networks minimizes competition and increases biodiversity. *Nature*.

[26] Joppa LN, Montoya JM, Solé R, Sanderson J, Pimm SL (2010). On nestedness in ecological networks. *Evolutionary Ecology Research*.

[27] Strona G, Galli P, Fattorini S (2013). Fish parasites resolve the paradox of missing coextinctions. *Nature Communications*.

[28] Ulrich W, Almeida-Neto M (2012). On the meanings of nestedness: back to the basics. *Ecography*.

[29] Blackburn TM, Brown VK, Doube BM, Greenwood JJD, Lawton JH, Stork NE (1993). The relationship between abundance and body size in natural animal assemblages. *Journal of Animal Ecology*.

[30] Guegan J-F, Kennedy CR (1993). Maximum local helminth parasite community richness in British freshwater fish: a test of the colonization time hypothesis. *Parasitology*.

[31] Strona G (2014). Assessing fish vulnerability: IUCN vs FishBase. *Aquatic Conservation: Marine and Freshwater Ecosystems*.

[32] Torchin ME, Lafferty KD, Dobson AP, McKenzie VJ, Kuris AM (2003). Introduced species and their missing parasites. *Nature*.

[33] Colautti RI, Ricciardi A, Grigorovich IA, MacIsaac HJ (2004). Is invasion success explained by the enemy release hypothesis?. *Ecology Letters*.

[34] Gendron AD, Marcogliese DJ, Thomas M (2012). Invasive species are less parasitized than native competitors, but for how long? The case of the round goby in the Great Lakes-St. Lawrence Basin. *Biological Invasions*.

[35] Mitchell CE, Power AO (2003). Release of invasive plants from fungal and viral pathogens. *Nature*.

[36] Blakeslee AMH, Altman I, Miller AW, Byers JE, Hamer CE, Ruiz GM (2012). Parasites and invasions: a biogeographic examination of parasites and hosts in native and introduced ranges. *Journal of Biogeography*.

[37] Lafferty KD, Torchin ME, Kuris AM, Morand S, Krasnov BR (2010). The geography of host and parasite invasions. *The Biogeography of Host—Parasite Interactions*.

[38] Roche DG, Leung B, Mendoza Franco EF, Torchin ME (2010). Higher parasite richness, abundance and impact in native versus introduced cichlid fishes. *International Journal for Parasitology*.

[39] Levin LA (2006). Recent progress in understanding larval dispersal: new directions and digressions. *Integrative and Comparative Biology*.

[40] Cribb TH, Pichelin S, Dufour V (2000). Parasites of recruiting coral reef fish larvae in New Caledonia. *International Journal for Parasitology*.

[41] Strona G, Fattorini S (2014). Parasitic worms: how many really?. *International Journal For Parasitology*.

[42] Strona G, Lafferty KD (2013). Predicting what helminth parasites a fish species should have using Parasite Co-Occurrence Modeler (PaCo). *Journal of Parasitology*.

[43] Guégan J-F, Kennedy CR (1996). Parasite richness/sampling effort/host range: the fancy three-piece jigsaw puzzle. *Parasitology Today*.

[44] Whittington ID (2004). The capsalidae (Monogenea: Monopisthocotylea): a review of diversity, classification and phylogeny with a note about species complexes. *Folia Parasitologica*.

[45] Rohde K (1993). *Ecology of Marine Parasites*.

[46] Kassen R (2002). The experimental evolution of specialists, generalists, and the maintenance of diversity. *Journal of Evolutionary Biology*.

[47] Clayton DH, Johnson KP (2003). Linking coevolutionary history to ecological process: doves and lice. *Evolution*.

[48] Kuris AM, Norton SF (1985). Evolutionary importance of overspecialization: insect parasitoids as an example. *The American Naturalist*.

[49] Strona G, Lafferty KD (2012). FishPEST: an innovative software suite for fish parasitologists. *Trends in Parasitology*.

[50] Strona G, Lafferty KD (2012). How to catch a parasite: Parasite Niche Modeler (PaNic) meets Fishbase. *Ecography*.

[51] Strona G, Palomares MLD, Bailly N, Galli P, Lafferty KD (2013). Host range, host ecology, and distribution of more than 11,800 fish parasite species. *Ecology*.

[52] Dove ADM (2006). Defining parasite communities is a challenge for neutral theory. *Journal of Parasitology*.

[53] Kuris AM, Blaustein AR, Alio JJ (1980). Hosts as islands. *The American Naturalist*.

[54] Seed JR, Sechelski JB (1996). The individual host, a unique evolutionary island for rapidly dividing parasites: a theoretical approach. *Journal of Parasitology*.

